# Supplementary material for: The evolutionary origin of host association in the Rickettsiales
Source: Nat Microbiol. 2022 Jul 7;7(8):1189–99. doi: 10.1038/s41564-022-01169-x (PMC9352585; doi:10.1038/s41564-022-01169-x)
Supplement: Supplementary file 1 — Supplementary Figs. 1–11, Text and Tables 1 and 2. [file 41564_2022_1169_MOESM1_ESM.pdf]

nature portfolio

https://doi.org/10.1038/s41564-022-01169-x

Supplementary information

The evolutionary origin of host association in the Rickettsiales

In the format provided by the authors and unedited

Supplementary Information for

The evolutionary origin of host association in the Rickettsiales

Max E. Schön, Joran Martijn, Julian Vosseberg, Stephan Köstlbacher and Thijs J. G. Ettema*

* Correspondence to: thijs.ettema@wur.nl

This PDF file includes:

Supplementary Text
Supplementary Figures 1-11
Supplementary Tables 1-2
Captions for Supplementary Data 1-6

Other Supplementary Information for this manuscript include the following:

Supplementary Data 1-6

1. Supplementary phylogenetic trees.
2. Sequencing runs of samples used for differential coverage binning.
3. Overview of genomes used for this study.
4. Annotation and presence/absence of selected gene families.
5. Results of the ancestral reconstruction using gene tree-species tree reconciliation.
6. Summary of the ancestral reconstruction.

Supplementary Text

Taxonomic Descriptions

Description of "Candidatus Mitibacter marchionensis*" (sp. nov, gen. nov.)*

Miti translates to "Sea" in Tahitian, close to the Marquesas Islands (latinised as marchionis), with suffix -ensis. Exclusively marine sequences, larger genomes than other Rickettsiales, flagellar and chemotaxis genes present, larger metabolic capacity (e.g. genetic repertoire for biosynthesis of all amino acids, nucleotides, etc.). 16S and 23S available.

Description of Candidatus Mitibacteraceae *(fam. nov.).*

Description is the same as for the genus Mitibacter, except that several MAGs were isolated from freshwater (UBA6178, UBA6189, UBA6149)[1,2]. Suff. -aceae, ending to denote a family. Type genus: Mitibacter

Description of Candidatus Athabascaceae *(fam. nov.).*

Prefix Athabasc- from the sampling location, the Athabasca oil sands syncrude tailing pond surface water near Fort McMurray in northeastern Alberta, Canada, from which the most complete MAG UBA6187 was extracted[1,3]. Also contains marine sequences (TARA_ANE_MAG_00011)[4]. Similarly to Mitibacteraceae, larger genomes than other Rickettsiales, flagellar and chemotaxis genes present, larger metabolic capacity (e.g. genetic repertoire for biosynthesis of all amino acids, nucleotides, etc.). No rRNA available. Suff. -aceae, ending to denote a family.

Description of Candidatus Gamibacteraceae *(fam. nov.).*

The word "Water" translates to //gam-i in Khoekhoe, a language of the Khoe languages, which are spoken by San and Khoekhoen people, native to South Africa. The MAGs C-132 and C-137 were both extracted from a marine sample that was taken close to the Cape region. Other MAGs were isolated from freshwater (UBA2645) and an aquifer (Alphaproteobacteria bacterium RIFCSPLOWO2 01 FULL 40 26)[1,5]. Reduced metabolic capacity (pathways for 18 amino acids present, nucleotide metabolism present), no flagellar or chemotaxis genes and no Tad system, no ATP/ADP translocase. 16S and 23S rRNA gene sequences are available. Suff. -aceae, ending to denote a family.

Phylogeny of the Gamibacteraceae and Deianireaeceae

The Alphaproteobacteria, of which the Rickettsiales are part of, are particularly sensitive to phylogenetic artefacts[6–9]. The generally AT-rich Rickettsiales often branch artifactually with other AT-rich alphaproteobacterial lineages such as Pelagibacterales, alpha proteobacterium HIMB59, MarineAlpha6, -7, -8 and -9 and the Holosporaceae. Although the phylogenetic relationships within the Rickettsiales are generally quite stable in the literature, there is some disagreement with respect to the placement of the Midichloriaceae. In some studies, they branch as sister to Rickettsiaceae[10,11], while in others they branch as sister to the Anaplasmataceae[11,12].

To test for the possible influence of compositional bias artefacts on the expanded Rickettsiales species tree, we inferred maximum likelihood and Bayesian phylogenies from an "untreated" supermatrix alignment and one that had the most heterogeneous sites removed via an iterative χ^2-trimming approach: here, all sites are ranked by their level of compositional heterogeneity as determined by a χ^2-score[8] and the top 1% most heterogeneous sites are removed. Then, per-site χ^2-scores are re-determined and the top 1% sites are removed again. In total, 47 such rounds were done (see Methods). The resulting trees were highly congruent, except for the placement of the Gamibacteraceae and Deianireaeceae. In the "untreated" trees, they branch as a clade sister to the Anaplasmataceae with near-maximum-to-maximum branch support (100 non-parametric bootstrap, NPB; 1.00 posterior probability, PP) and in the "iterative χ^2-trimmed" trees, they branch as as sister to a clade comprising Midichloriaceae and Anaplasmataceae, highly supported in the bayesian phylogeny (0.99 PP), but lacking support in the ML tree (53 NPB) . This suggests that the placement of the Gamibacteraceae and Deianiraeaceae clade sister to Anaplasmataceae (also observed in[13]) may be the result of a phylogenetic artefact.

Putative T4SS effectors in Mitibacteraceae and Athabascaceae

While no verified Rickettsiales effector proteins seem to be conserved across families, many of the experimentally verified effectors contain eukaryotic-like repeat domains such as ankyrin repeat (ANK), leucine rich repeat (LRR) or tetratricopeptide repeat (TPR)[14–19]. Proteins with such domains are numerous in the Rickettsiales *Orientia tsutsugamushi*[16,20] (46 ANK, 22 TPR) and *Ca.* Jidaibacter acanthamoeba (131 ANK, 12 LRR, 59 TPR). However, we were unable to detect a large number of such proteins in the Mitibacteraceae (max. 5 ANK, 16 TPR) and Athabascaceae (max. 23 ANK, 5 LRR, 29 TPR) genomes (Extended Data Figure 4). Other potential effector proteins may have escaped detection as they are known to be highly taxon specific and rarely conserved over larger evolutionary distances[21]. Interestingly, we could not identify many potential effectors with such domains in the recently described *Ca.* D. vastatrix (2 ANK, 5 TPR) and many obligate intracellular Rickettsiales either, indicating that most identified effectors are perhaps very taxon specific (Extended Data Figure 5, Supplementary Data 4). Furthermore, the presence of these domains is by itself not necessarily indicative of host-associated effector proteins, and these proteins could be involved in other functions as well[22]. Instead, we could identify several domains that have been identified in effector proteins of the bacteria-killing T4SS of *Xanthomonas citri*, such as peptidoglycan binding domains (PGBD; Mitibacteraceae: max. 6; Athabascaceae: max. 2; other Rickettsiales: max. 2) and Peptidase M23 (Mitibacteraceae: max. 8; Athabascaceae: max. 4; other Rickettsiales: max. 5)[23,24]. Furthermore, we could trace four copies of proteins with such PGBD in three clusters (01QGY, 01RY1 and 01R8F; Supplementary Data 5) to the LRCA.

Supplementary References

1. Parks, D. H. *et al.* Recovery of nearly 8,000 metagenome-assembled genomes substantially expands the tree of life. *Nat. Microbiol.* (2017) doi:10.1038/s41564-017-0012-7.

2. Tully, B. J., Graham, E. D. & Heidelberg, J. F. The reconstruction of 2,631 draft

metagenome-assembled genomes from the global oceans. *Sci. Data* **5**, 170203 (2018).

3. Saidi-Mehrabad, A. *et al.* Methanotrophic bacteria in oilsands tailings ponds of northern Alberta. *ISME J.* **7**, 908–921 (2013).
4. Delmont, T. O. *et al.* Nitrogen-fixing populations of Planctomycetes and Proteobacteria are abundant in surface ocean metagenomes. *Nat. Microbiol.* **3**, 804–813 (2018).
5. Anantharaman, K. *et al.* Thousands of microbial genomes shed light on interconnected biogeochemical processes in an aquifer system. *Nat. Commun.* **7**, 13219 (2016).
6. Martijn, J., Vosseberg, J., Guy, L., Offre, P. & Ettema, T. J. G. Deep mitochondrial origin outside the sampled alphaproteobacteria. *Nature* **557**, 101–105 (2018).
7. Rodríguez-Ezpeleta, N. & Embley, T. M. The SAR11 Group of Alpha-Proteobacteria Is Not Related to the Origin of Mitochondria. *PLOS ONE* **7**, e30520 (2012).
8. Viklund, J., Ettema, T. J. G. & Andersson, S. G. E. Independent Genome Reduction and Phylogenetic Reclassification of the Oceanic SAR11 Clade. *Mol. Biol. Evol.* **29**, 599–615 (2012).
9. Muñoz-Gómez, S. A. *et al.* An updated phylogeny of the Alphaproteobacteria reveals that the parasitic Rickettsiales and Holosporales have independent origins. *eLife* **8**, e42535 (2019).
10. Sassera, D. *et al.* Phylogenomic Evidence for the Presence of a Flagellum and cbb3 Oxidase in the Free-Living Mitochondrial Ancestor. *Mol. Biol. Evol.* **28**, 3285–3296 (2011).
11. Driscoll, T., Gillespie, J. J., Nordberg, E. K., Azad, A. F. & Sobral, B. W. Bacterial DNA Sifted from the Trichoplax adhaerens (Animalia: Placozoa) Genome Project Reveals a Putative Rickettsial Endosymbiont. *Genome Biol. Evol.* **5**, 621–645 (2013).
12. Montagna, M. *et al.* ‘Candidatus Midichloriaceae’ fam. nov. (Rickettsiales), an ecologically

widespread clade of intracellular alphaproteobacteria. *Appl. Environ. Microbiol.* **79**, 3241–3248 (2013).

13. Castelli, M. *et al.* Deianiraea, an extracellular bacterium associated with the ciliate Paramecium, suggests an alternative scenario for the evolution of Rickettsiales. *ISME J.* **13**, 2280–2294 (2019).
14. Gillespie, J. J. *et al.* Secretome of obligate intracellular Rickettsia. *FEMS Microbiol. Rev.* **39**, 47–80 (2015).
15. Lockwood, S. *et al.* Identification of Anaplasma marginale Type IV Secretion System Effector Proteins. *PLOS ONE* **6**, e27724 (2011).
16. Schulz, F. *et al.* A Rickettsiales symbiont of amoebae with ancient features. *Environ. Microbiol.* **18**, 2326–2342 (2016).
17. Rennoll-Bankert, K. E. *et al.* Which Way In? The RalF Arf-GEF Orchestrates Rickettsia Host Cell Invasion. *PLoS Pathog.* **11**, e1005115 (2015).
18. Lehman, S. S. *et al.* The Rickettsial Ankyrin Repeat Protein 2 Is a Type IV Secreted Effector That Associates with the Endoplasmic Reticulum. *mBio* **9**, e00975-18 (2018).
19. Voss, O. H. *et al.* Risk1, a Phosphatidylinositol 3-Kinase Effector, Promotes Rickettsia typhi Intracellular Survival. *mBio* **11**, e00820-20 (2020).
20. Cho, N.-H. *et al.* The Orientia tsutsugamushi genome reveals massive proliferation of conjugative type IV secretion system and host–cell interaction genes. *Proc. Natl. Acad. Sci.* **104**, 7981–7986 (2007).
21. Gillespie, J. J. *et al.* An Anomalous Type IV Secretion System in Rickettsia Is Evolutionarily Conserved. *PLOS ONE* **4**, e4833 (2009).
22. Al-Khodor, S., Price, C. T., Kalia, A. & Kwaik, Y. A. Ankyrin-repeat containing proteins of

microbes: a conserved structure with functional diversity. *Trends Microbiol.* **18**, 132–139 (2010).

23. Souza, D. P. *et al.* Bacterial killing via a type IV secretion system. *Nat. Commun.* **6**, 6453 (2015).

24. Sgro, G. G. *et al.* Bacteria-Killing Type IV Secretion Systems. *Front. Microbiol.* **10**, (2019).

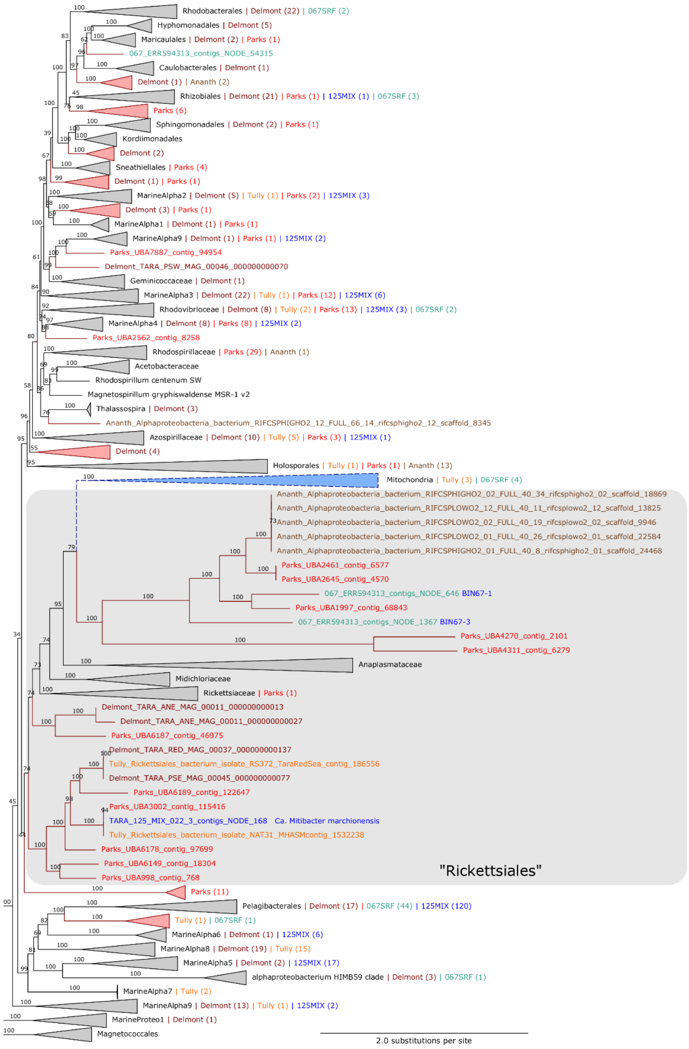

Supplementary Figure 1. Alphaproteobacterial and mitochondrial phylogenetic diversity in analysed metagenomic datasets. Phylogenetic analyses of concatenated ribosomal protein sequences (see Methods) identified in the metagenome assemblies of the two Tara Ocean's environmental samples 'TARA_125_MIX_022-3' (dark blue) and 'TARA_067_SRF_0.22-0.45' (teal) and the metagenome assembled genomes of Tully *et al* (*43*) (orange), Parks *et al* (*42*) (red), Delmont *et al* (*44*) (brown-red) and Anantharaman *et al* (*45*) (light-brown). Reference taxa are given in black. Clades are collapsed in grey triangles when they include reference taxa and in red triangles when they include no reference taxa. The number of environmental alphaproteobacteria and mitochondria is denoted in the collapsed triangle annotation. Length of mitochondrial stem branch is shortened to improve tree readability. Tree was reconstructed with the RP15 pipeline (see Methods). The complete tree is available as a Newick file (Supplementary Data 1).

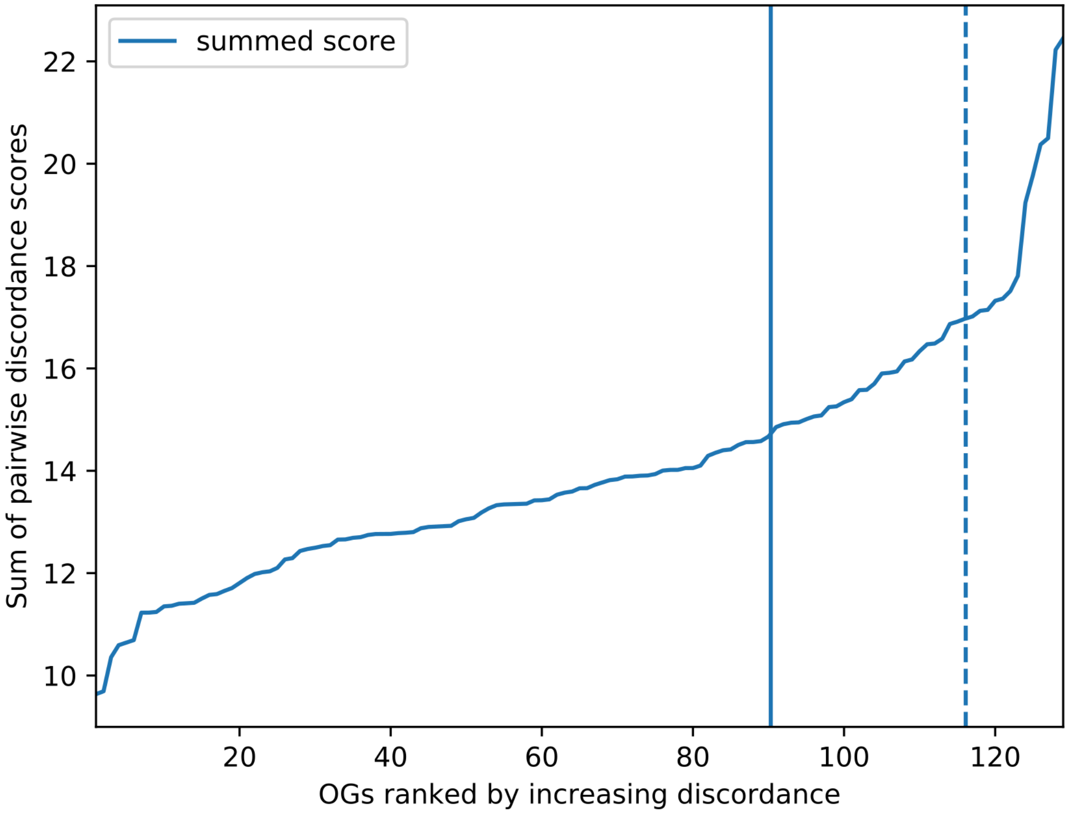

Supplementary Figure 2. Discordance filter of the 129 panortholog dataset from Martijn et al. (*25*)**.** Bipartition count profiles were constructed from the bootstraps of each single gene tree and compared between all possible gene pairs to calculate discordance scores. Genes were then ordered according to increasing discordance. The 13 most discordant genes, scoring above the dotted line, were rejected.

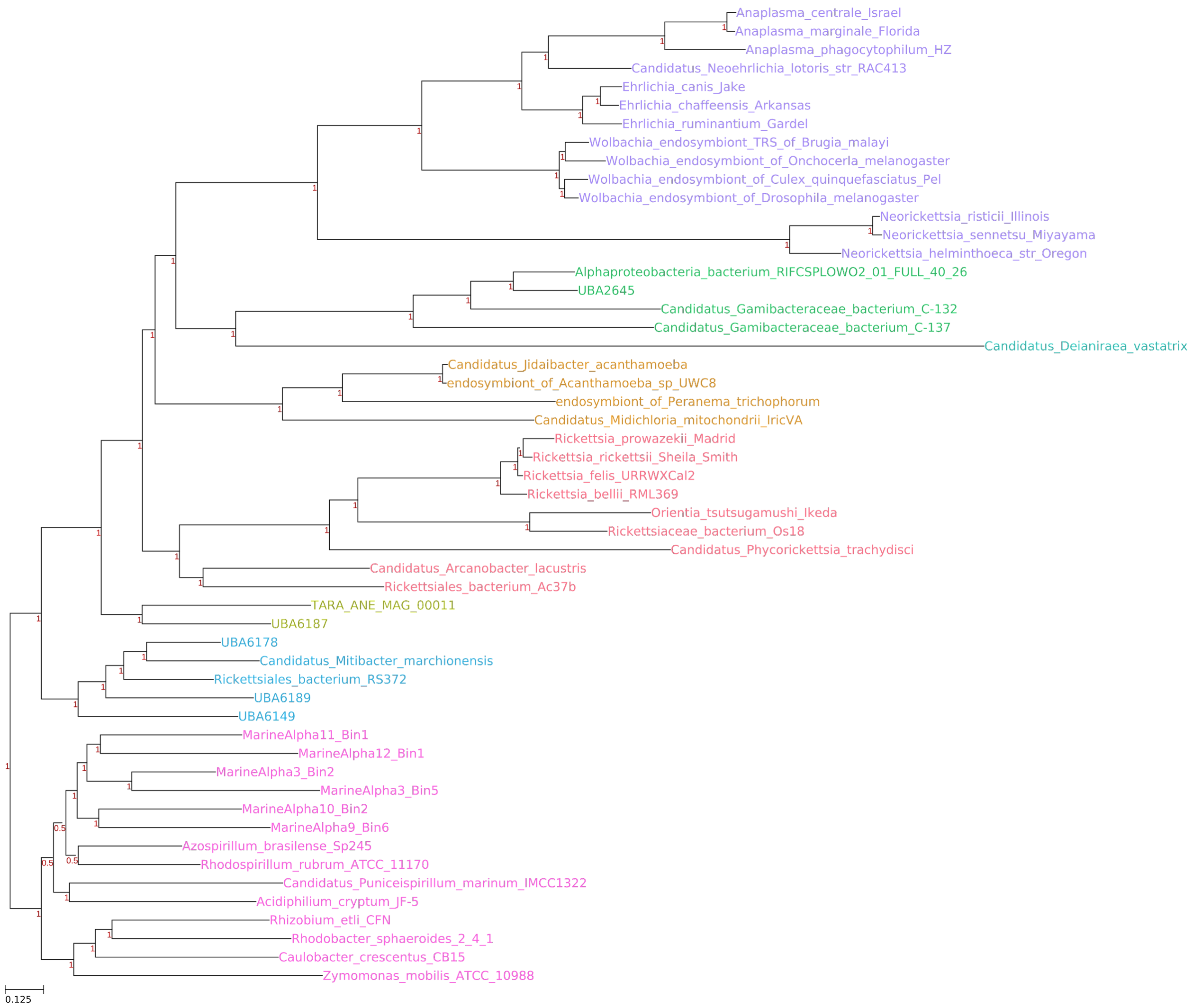

Supplementary Figure 3. Bayesian inference of Rickettsiales phylogeny based on the untreated concatenated alignment of 116 panorthologs. Consensus tree over all 4 MCMC chains that were inferred under the CAT+GTR+Γ4 model is given. Anaplasmataceae (purple), Gamibacteraceae (green), Deianiraea (teal), Midichloriaceae (orange), Rickettsiaceae (salmon), Athabascaceae (moss), Mitibacteraceae (blue) and other alphaproteobacteria (pink). Support values (in red) correspond to posterior probabilities. The four trees converged with respect to the in-group, but failed to converge with respect to the outgroups. Consensus trees of the individual MCMC chains are available as Newick files (Data 1).

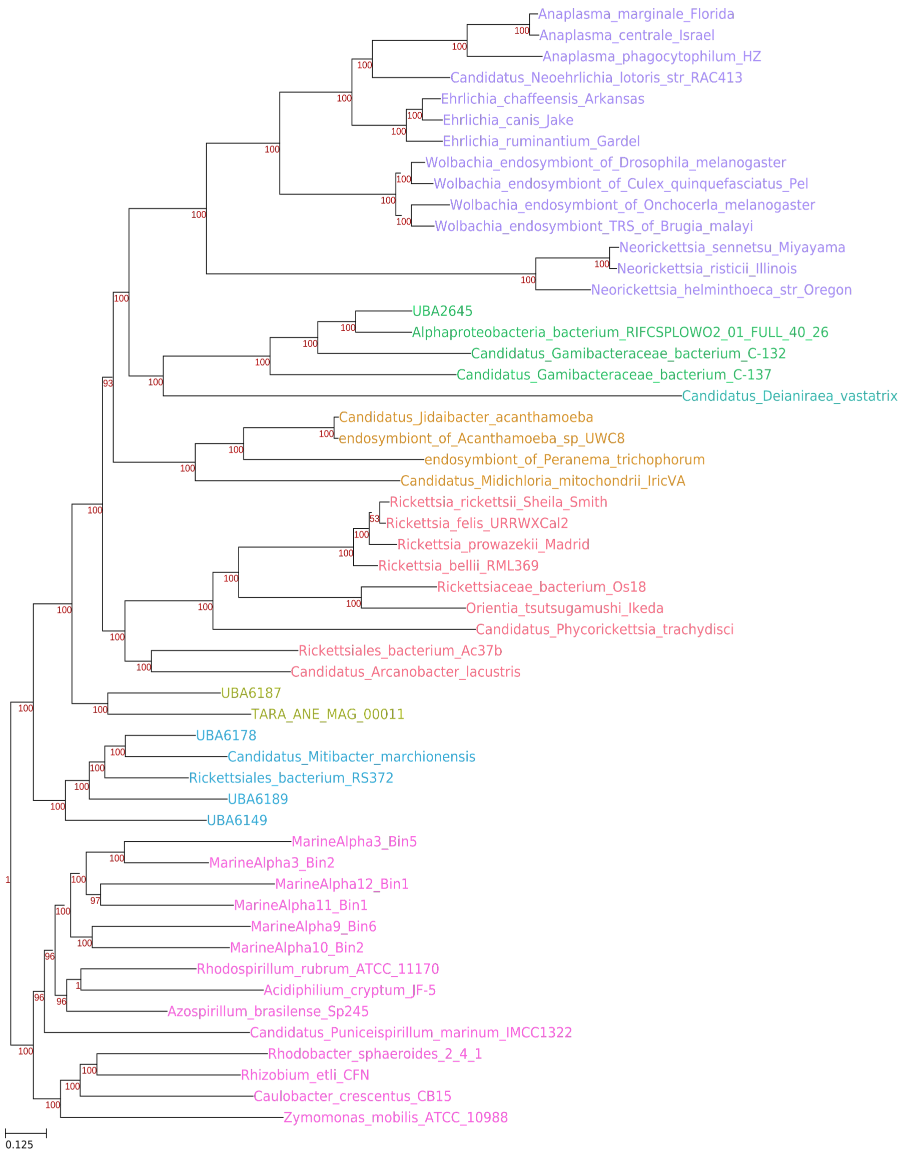

Supplementary Figure 4. Maximum likelihood phylogeny of Rickettsiales based on the untreated concatenated alignment of 116 panorthologs. ML tree inferred under the PMSF approximation of LG+C60+F+Γ4 with 100 non-parametric bootstraps as implemented by IQTREE. Anaplasmataceae (purple), Gamibacteraceae (green), Deianiraea (teal), Midichloriaceae (orange), Rickettsiaceae (salmon), Athabascaceae (moss), Mitibacteraceae (blue) and other alphaproteobacteria (pink).

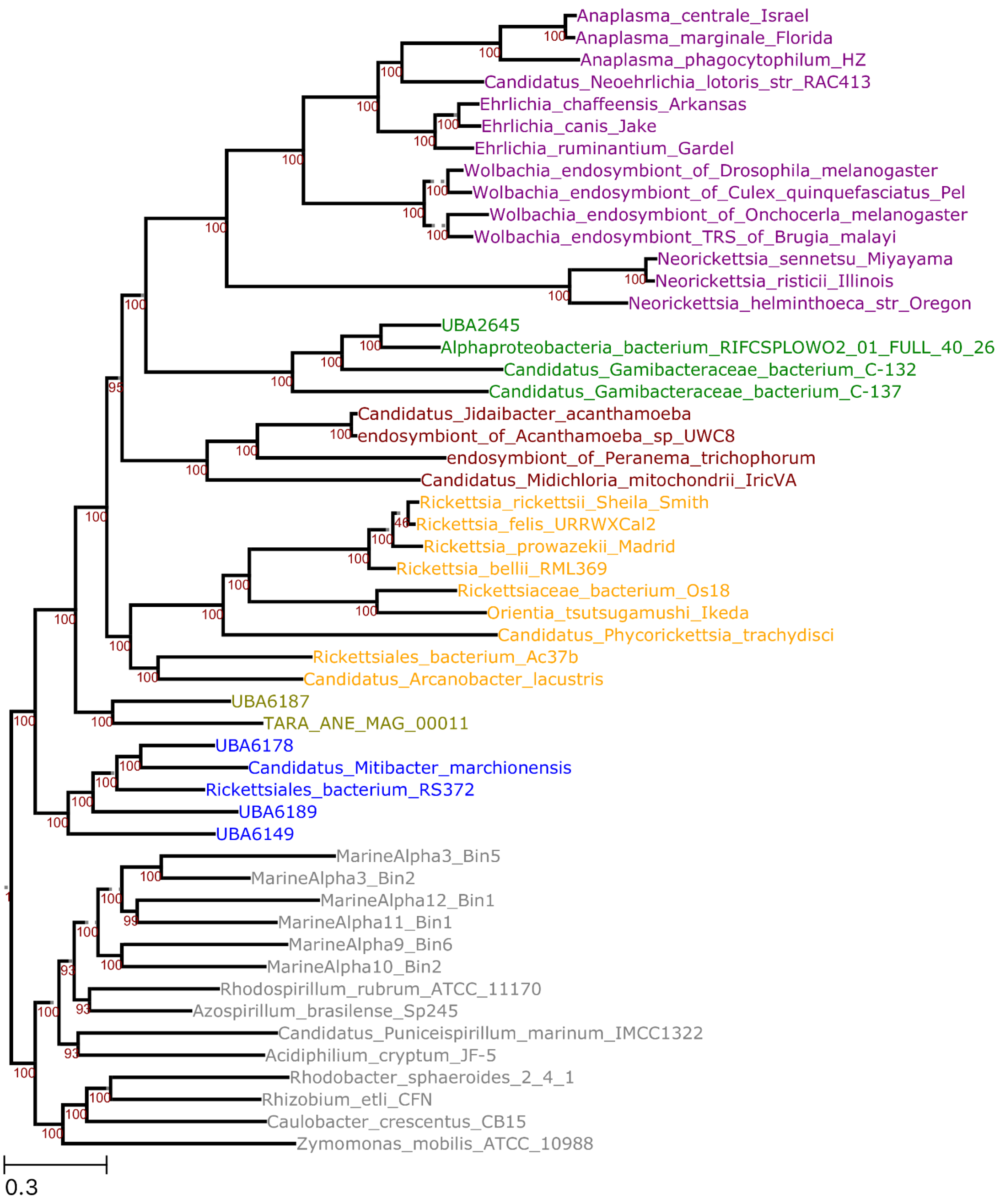

Supplementary Figure 5. Maximum likelihood phylogeny of Rickettsiales based on the untreated concatenated alignment of 116 panorthologs, excluding Deianiraea. ML tree inferred under the PMSF approximation of LG+C60+F+Γ4 with 100 non-parametric bootstraps as implemented by IQTREE. Anaplasmataceae (purple), Gamibacteraceae (green), Midichloriaceae (dark red), Rickettsiaceae (yellow), Athabascaceae (moss), Mitibacteraceae (blue) and other alphaproteobacteria (grey).

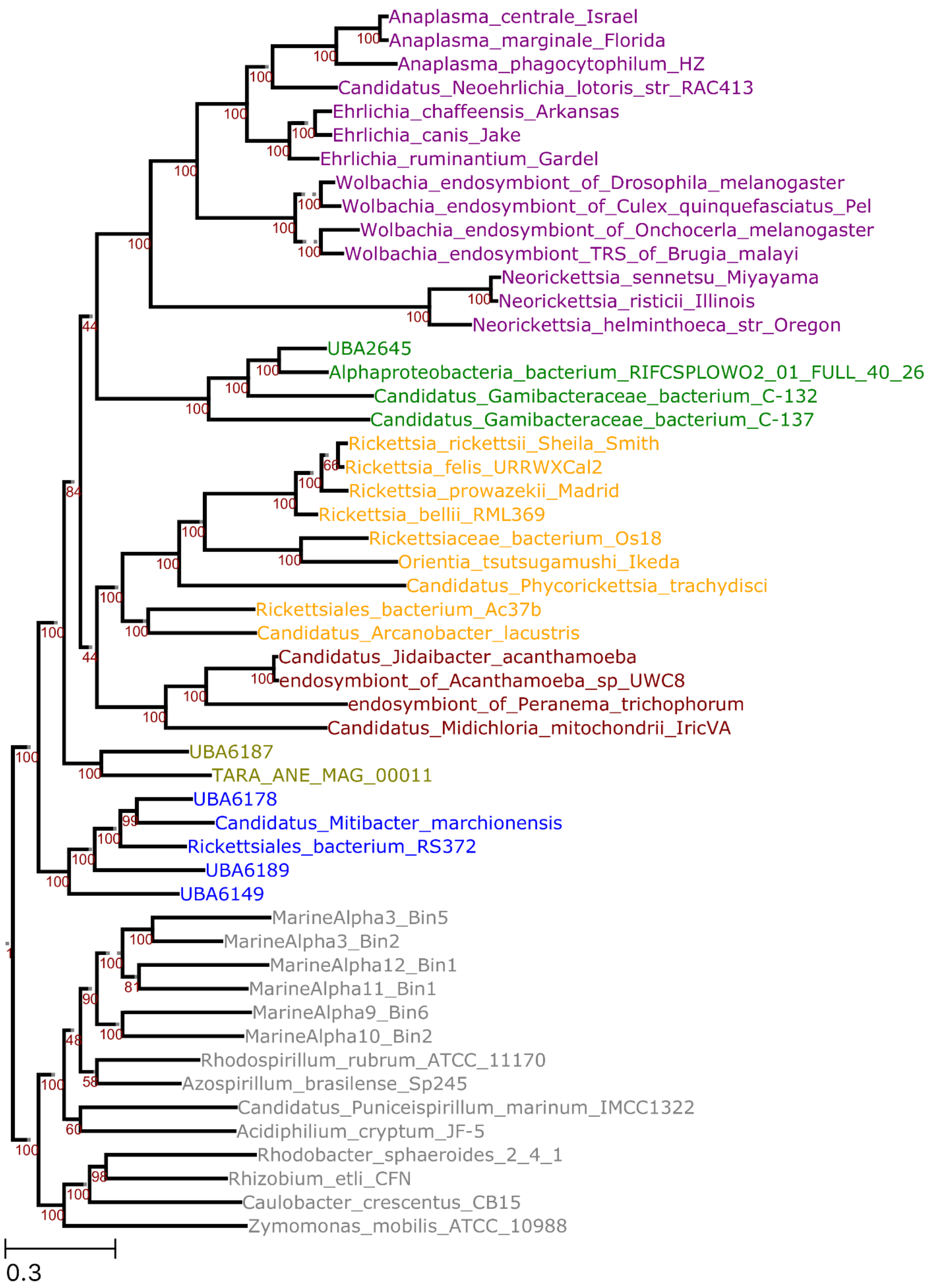

Supplementary Figure 6. Maximum likelihood phylogeny of Rickettsiales based on an concatenated alignment of 116 panorthologs with the 40% most heterogeneous sites removed and excluding Deianiraea. The alignment was prepared using χ^2-trimming: the top 40% most heterogeneous sites as determined by χ^2-score were removed. ML tree inferred under the PMSF approximation of LG+C60+F+Γ4 with 100 non-parametric bootstraps as implemented by IQTREE. Anaplasmataceae

(purple), Gamibacteraceae (green), Midichloriaceae (dark red), Rickettsiaceae (yellow), Athabascaceae (moss), Mitibacteraceae (blue) and other alphaproteobacteria (grey).

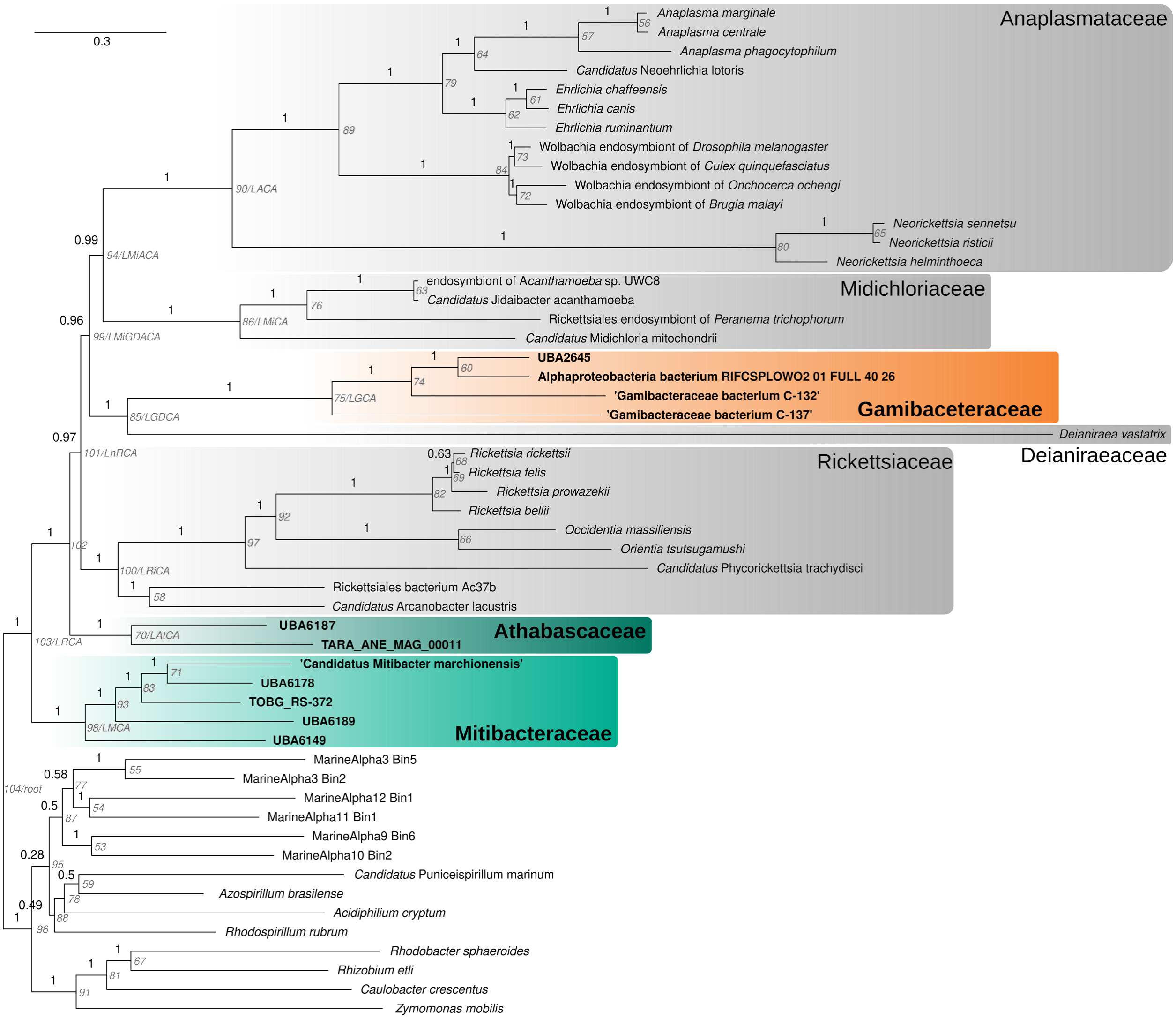

Supplementary Figure 7. Bayesian inference Rickettsiales species tree with alphaproteobacterial outgroup based on a dataset of 116 marker genes. The alignment was prepared using iterative χ^2-trimming: 47 rounds of removing the top 1% most heterogeneous sites as determined by χ^2-score), highlighting the phylogenetic position of Gamibacteraceae (orange shading), Athabascaceae (dark green shading) and Mitibacteraceae (light green shading). The tree was inferred with PhyloBayes (CAT+LG+Γ4, 20000 cycles with a burn-in of 5000 cycles). Support values (in black) correspond to posterior probabilities. Internal node names and ancestor designations used for the ancestral reconstruction are written in grey. Last common ancestor of Anaplasmataceae, Midichloriaceae, Deianiraea and Gamibacteraceae: LMiGDACA; Last common ancestor of Anaplasmataceae and Midichloriaceae: LMiACA; Last common ancestor of Deianiraea and Gamibacteraceae: LGDCA; Last common ancestor of Anaplasmataceae: LACA, Midichloriaceae: LMiCA, Gamibacteraceae: LGCA; Rickettsiaceae: LRiCA, Mitibacteraceae: LMCA, Athabascaceae: LAtCA.

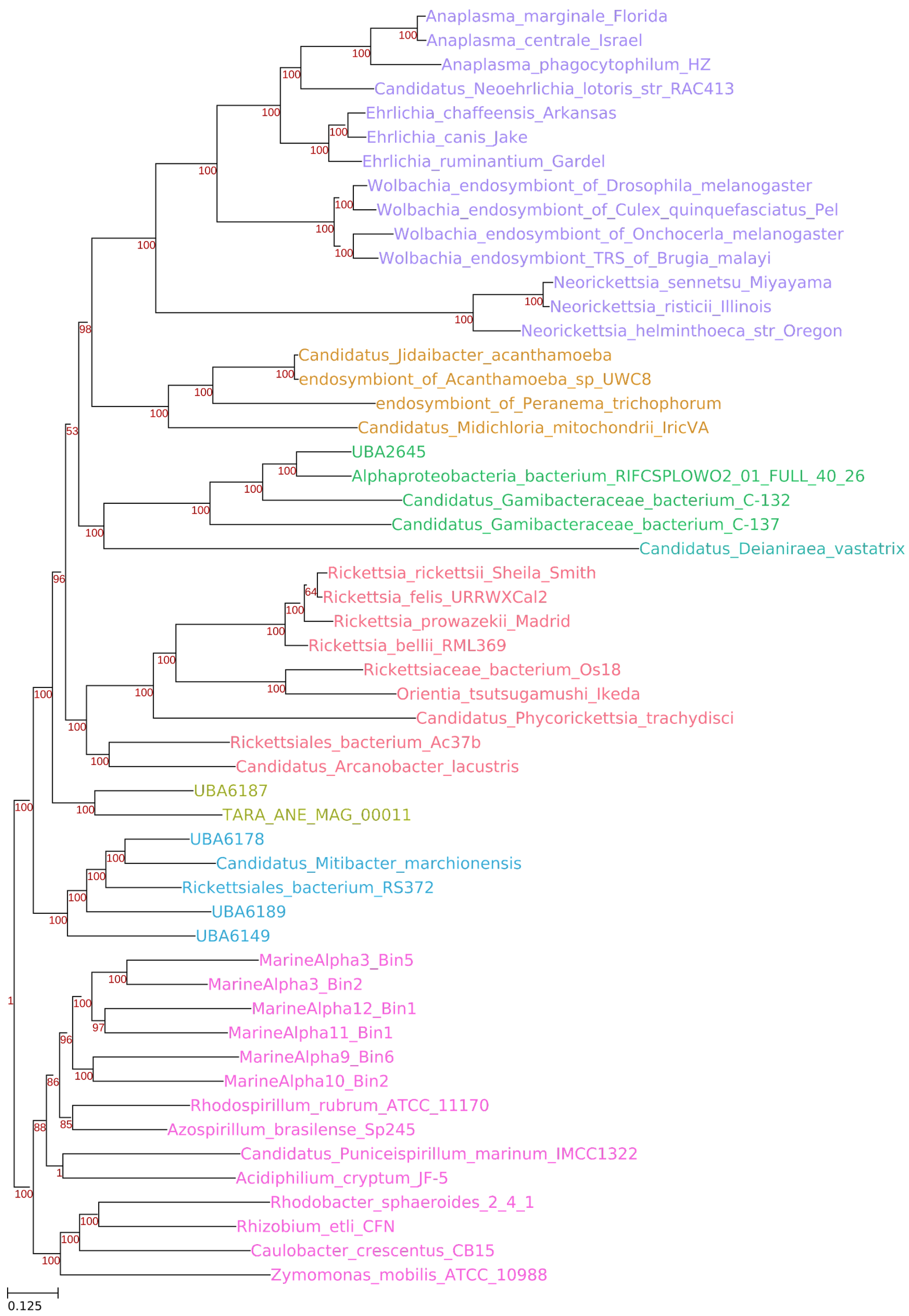

Supplementary Figure 8. Maximum likelihood Rickettsiales species tree with alphaproteobacterial outgroup based on a dataset of 116 marker genes. The alignment was prepared using iterative χ^2-trimming: 47 rounds of removing the top 1% most heterogeneous sites as determined by χ^2-score. ML tree inferred under the PMSF approximation of LG+C60+F+Γ4 with 100 non-parametric bootstraps as implemented by IQTREE. Anaplasmataceae (purple), Gamibacteraceae (green), Deianiraea (teal),

Midichloriaceae (orange), Rickettsiaceae (salmon), Athabascaceae (moss), Mitibacteraceae (blue) and other alphaproteobacteria (pink).

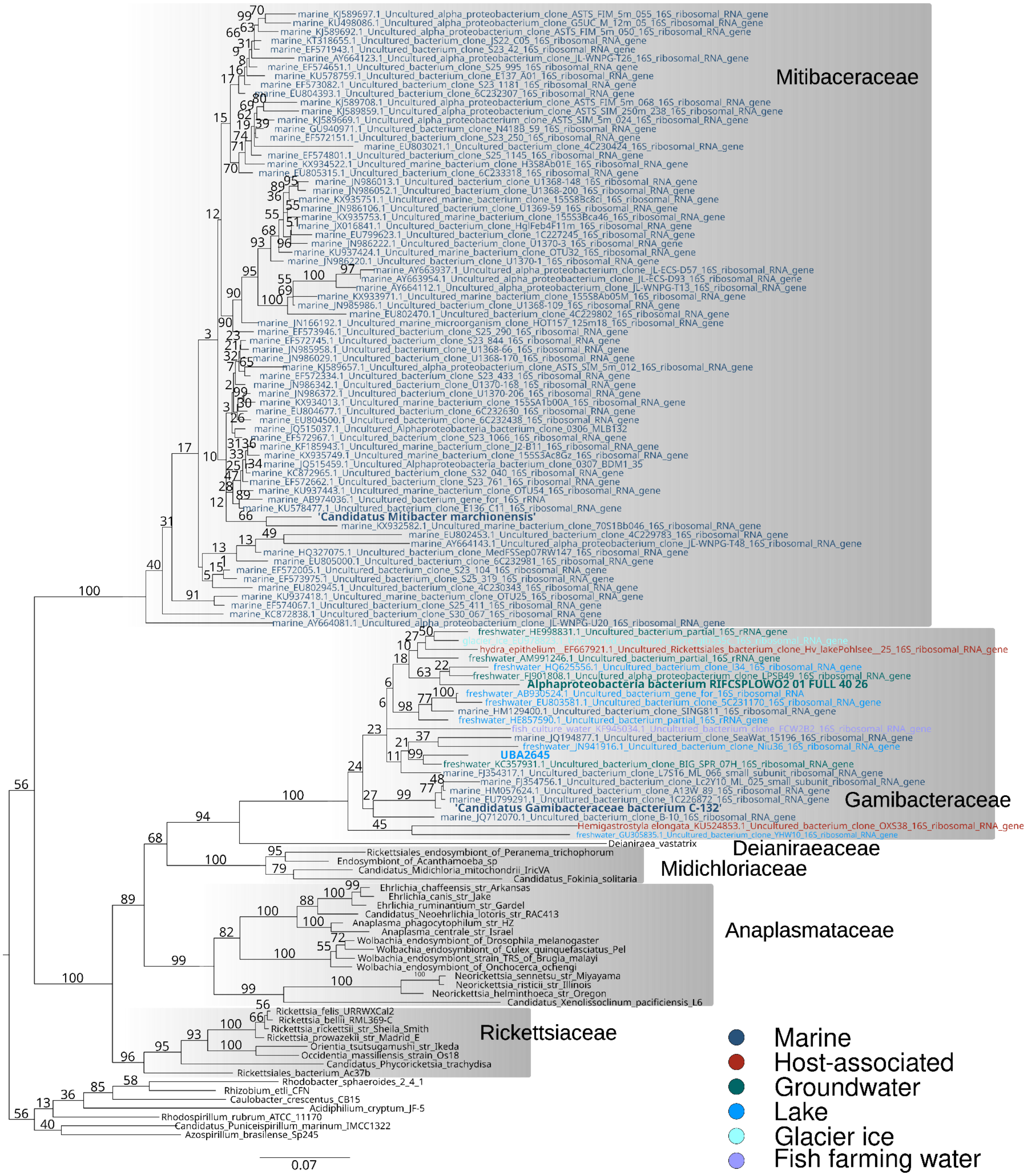

Supplementary Figure 9. Phylogenetic tree of 16S rRNA gene sequences related to Mitibacteraceae and Gamibacteraceae. Sequences of environmental origin are color-coded (see legend) and reference sequences from other Rickettsiales families and an alphaproteobacterial outgroup are indicated. Support

values correspond to 100 non-parametric bootstraps. The tree was reconstructed in IQ-Tree under the GTR+F+R8 model (see Methods).

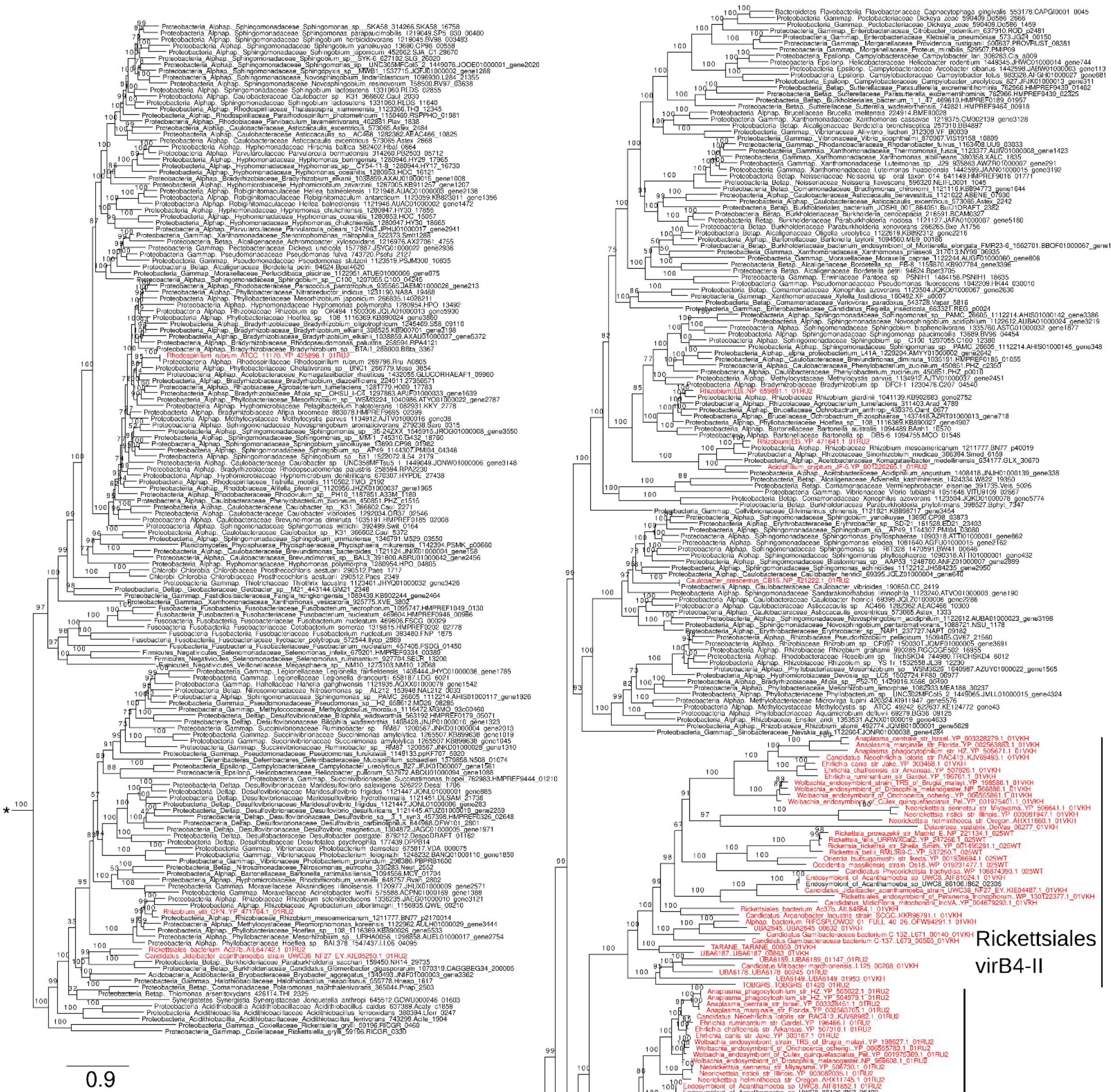
0.9
Rickettsiales
virB4-II
Rickettsiales
virB4-I

Supplementary Figure 10. Phylogenetic tree of virB4 homologs from the sampled Rickettsiales genomes and reference sequences from the EggNOG v5.0 database (COG3451). Tree was reconstructed in IQ-TREE under the model LG+F+R10 with 1000 ultrafast bootstraps. Taxa included in the ancestral reconstruction are highlighted in red, reference sequences include a taxonomic label. Rickettsiales virB4-1 and virB4-2 are monophyletic, suggesting a common duplication in LRCA. The two subtrees are connected at the edges labelled with '*'.

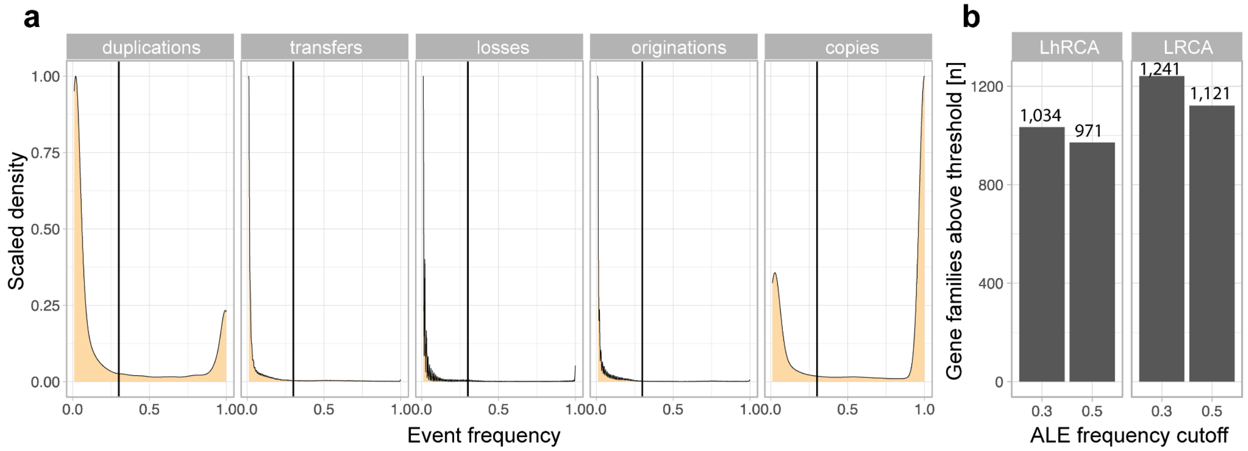

Supplementary Figure 11. Density distribution of ALE event relative frequencies and impact of cutoffs on reconstructed Rickettsiales ancestor gene content. (a) Plot displaying the scaled density distribution of relative frequencies of inferred ALE events on the y-axis and x-axis, respectively. Vertical lines indicate the selected relative frequency cutoff of 0.3. The number of evolutionary events inferred by ALE follow a bimodal distribution, indicating either a very strong signal for their occurrence (close to 1) or very strong signal for absence (close to 0), with most low frequency events occurring below the selected cutoff. (b) Bar plot representing the number of inferred gene families (y-axis) with relative frequencies above the cutoffs 0.3 or 0.5 (x-axis) for the last common ancestors of the classical, obligate host-associated Rickettsiales (LhRCA) and all Rickettsiales (LRCA), respectively.

Supplementary Table 1. Overview of Bayesian phylogenetic analyses. Summary statistics of alignments, MCMC sampling and posterior predictive tests (one-sided) of Bayesian phylogenetic analyses.

**Dataset: 116 near-panorthologs Rickettsiales**	**Untreated**	**iterative χ2-trim *(47 rounds of removing the top 1% sites)***
Sites	38842	24238
Informative sites	29598	15347
Missing data (%)	21.3	24.1
χ2-score	78360	423
Model of evolution	CAT+GTR+Γ4	CAT+LG+Γ4
MCMC sampling statistics		
Generations	32000	20100
Burnin	15000	5000
Effective sample size*	>533	>240
Maxdiff	1	1
*Posterior predictive tests***		
Maximum squared heterogeneity (p-value)	0	0.87 - 0.90
Maximum squared heterogeneity (Z-score)	240 - 278	-1.1 - -1.1
Mean squared heterogeneity (p-value)	0	0.90 - 0.94
Mean squared heterogeneity (Z-score)	966 - 1075	-1.3 - -1.4
Mean diversity per site (p-value)	0.01 - 0.04	0.04 - 0.06
Mean diversity per site (Z-score)	2.0 - 2.1	1.5 - 1.7
* Considering only log-likelihood, total tree length, alpha parameter and number of categories		
** Range across 4 chains		

Supplementary Table 2. Overview of obligate intracellular lifestyle predictions. Rickettsiales genome representative with their family rank, PhenDB obligate intracellular lifestyle prediction (+), different lifestyle (-). "n.d." indicates confidence < 0.6.

Genome	Family	Obligate intracell. lifestyle	Prediction confidence
Anaplasma_centrale_Israel	Anaplasmataceae	+	0.91
Anaplasma_marginale_Florida	Anaplasmataceae	+	0.91
Anaplasma_phagocytophilum_HZ	Anaplasmataceae	+	0.92
Candidatus_Neoehrlichia_lotoris_str_RAC413	Anaplasmataceae	+	0.9
Ehrlichia_canis_Jake	Anaplasmataceae	+	0.91
Ehrlichia_chaffeensis_Arkansas	Anaplasmataceae	+	0.91
Ehrlichia_ruminantium_Gardel	Anaplasmataceae	+	0.91
Neorickettsia_helminthoeca_str_Oregon	Anaplasmataceae	+	0.89
Neorickettsia_risticii_Illinois	Anaplasmataceae	+	0.92
Neorickettsia_sennetsu_Miyayama	Anaplasmataceae	+	0.92
Wolbachia_endosymbiont_of_Culex_quinquefasciatus_Pel	Anaplasmataceae	+	0.93
Wolbachia_endosymbiont_of_Drosophila_melanogaster	Anaplasmataceae	+	0.93
Wolbachia_endosymbiont_of_Onchocerla_melanogaster	Anaplasmataceae	+	0.93
Wolbachia_endosymbiont_TRS_of_Brugia_malayi	Anaplasmataceae	+	0.94
Candidatus_Jidaibacter_acanthamoeba	Midichloriaceae	+	0.9
Candidatus_Midichloria_mitochondrii_IricVA	Midichloriaceae	+	0.93
endosymbiont_of_Acanthamoeba_sp_UWC8	Midichloriaceae	+	0.91
Rickettsiales_endosymbiont_of_Peranema_trichophorum	Midichloriaceae	+	0.87
Deianiraea_vastatrix	Deiniraeaceae	+	0.81
Alphaproteobacteria_bacterium_RIFCSPLOWO2_01_FULL_40_26	Gamibacteraceae	n.d.	0.58
bin_67_1	Gamibacteraceae	n.d.	0.53
bin_67_3	Gamibacteraceae	n.d.	0.5
UBA2645	Gamibacteraceae	-	0.69
Candidatus_Arcanobacter_lacustris	Rickettsiaceae	+	0.72
Candidatus_Phycoricketsia_trachydisa	Rickettsiaceae	+	0.93
Orientia_tsutsugamushi_Ikeda	Rickettsiaceae	+	0.95
Rickettsia_bellii_RML369	Rickettsiaceae	+	0.94
Rickettsia_felis_URRWXCal2	Rickettsiaceae	+	0.93
Rickettsia_prowazekii_Madrid	Rickettsiaceae	+	0.94
Rickettsia_rickettsii_Sheila_Smith	Rickettsiaceae	+	0.93
Rickettsiaceae_bacterium_Os18	Rickettsiaceae	+	0.94
Rickettsiales_bacterium_Ac37b	Rickettsiaceae	+	0.85
TARA_ANE_MAG_00011	Athabascaceae	-	0.73
UBA6187	Athabascaceae	-	0.66
bin_125	Mitibacteraceae	-	0.72
TOBG_RS-372	Mitibacteraceae	-	0.69
UBA6149	Mitibacteraceae	-	0.69
UBA6178	Mitibacteraceae	-	0.7
UBA6189	Mitibacteraceae	-	0.74

Supplementary Data 1. (separate file)
Supplementary phylogenetic trees. Tar archive with all phylogenetic trees (rp15, alphamito24 and the 116 gene dataset) showing the position of the novel MAGs in newick format.

Supplementary Data 2. (separate file)
Sequencing runs of samples used for differential coverage binning. Samples that were assembled and of which the contigs were binned are highlighted in orange. All samples were used for differential coverage binning of TARA_125_SRF_0.22-3. Samples highlighted in green and ERR594313 were used for differential coverage binning of TARA_067_SRF_0.22-0.45. This table is a subset of the companion Table W1 published by Sunagawa et al, 2015 (*37*).

Supplementary Data 3. (separate file)
Overview of genomes used for this study. Assembly statistics (overall size, number of contigs, N50, estimated completeness) and important genomic characteristics (G+C content, coding density number of CDS) as well as the clade assignment are presented.

Supplementary Data 4. (separate file)
Annotation and presence/absence of selected gene families. Annotation of gene families related to selected biochemical processes are presented, with number of genes as found in the selected Rickettsiales and alphaproteobacterial taxa as well as inferred for the ancestors of Rickettsiales (LRCA), host-associated Rickettsiales (LhRCA) and all Rickettsiales families. Last common ancestor of Anaplasmataceae, Midichloriaceae, Deianiraea and Gamibacteraceae: LMiGDACA; Last common ancestor of Anaplasmataceae and Midichloriaceae: LMiACA; Last common ancestor of Anaplasmataceae: LACA, Midichloriaceae: LMiCA, Gamibacteraceae: LGCA; Rickettsiaceae: LRiCA, Mitibacteraceae: LMCA, Athabascaceae: LAtCA.

Supplementary Data 5. (separate file)
Results of the ancestral reconstruction using gene tree-species tree reconciliation. Inferences by ALEml_undated for gene family presence/absence in ancestral and extant lineages, with a general threshold of 0.3 applied.

Supplementary Data 6. (separate file)
Summary of the ancestral reconstruction. Inferences by ALEml_undated for ancestral and extant gene family numbers as well as estimated numbers of originations, duplications, transfers and losses per node. Summary of Supplementary Data 5.

Figshare repository (10.6084/m9.figshare.c.5494977):

- All protein clusters fasta format, proteins alignments, phylogenetic trees generated in the present study
- Vir subunits phylogenetic trees in nexus and pdf format
- Raw ancestral genome reconstruction (ALE) results
- Effector domain profile search (hmmsearch) output
- Protein-level annotations
