# Supplementary material for: The evolutionary origin of host association in the Rickettsiales
Source: Nat Microbiol. 2022 Jul 7;7(8):1189–99. doi: 10.1038/s41564-022-01169-x (PMC9352585; doi:10.1038/s41564-022-01169-x)
Supplement: Supplementary file 2 — Reporting Summary [file 41564_2022_1169_MOESM2_ESM.pdf]

Corresponding author(s): Ettema

Last updated by author(s): 24-05-2022

Reporting Summary

Nature Portfolio wishes to improve the reproducibility of the work that we publish. This form provides structure for consistency and transparency in reporting. For further information on Nature Portfolio policies, see our Editorial Policies and the Editorial Policy Checklist.

Statistics

For all statistical analyses, confirm that the following items are present in the figure legend, table legend, main text, or Methods section.

n/a	Confirmed	
☐	☒	The exact sample size (*n*) for each experimental group/condition, given as a discrete number and unit of measurement
☒	☐	A statement on whether measurements were taken from distinct samples or whether the same sample was measured repeatedly
☒	☐	The statistical test(s) used AND whether they are one- or two-sided<br>*Only common tests should be described solely by name; describe more complex techniques in the Methods section.*
☒	☐	A description of all covariates tested
☒	☐	A description of any assumptions or corrections, such as tests of normality and adjustment for multiple comparisons
☒	☐	A full description of the statistical parameters including central tendency (e.g. means) or other basic estimates (e.g. regression coefficient) AND variation (e.g. standard deviation) or associated estimates of uncertainty (e.g. confidence intervals)
☐	☒	For null hypothesis testing, the test statistic (e.g. *F*, *t*, *r*) with confidence intervals, effect sizes, degrees of freedom and *P* value noted<br>*Give P values as exact values whenever suitable.*
☒	☐	For Bayesian analysis, information on the choice of priors and Markov chain Monte Carlo settings
☒	☐	For hierarchical and complex designs, identification of the appropriate level for tests and full reporting of outcomes
☒	☐	Estimates of effect sizes (e.g. Cohen's *d*, Pearson's *r*), indicating how they were calculated

Our web collection on statistics for biologists contains articles on many of the points above.

Software and code

Policy information about availability of computer code

Data collection	No software was used for data collection
Data analysis	SEQPREP v1.3.2, TRIMMOMATIC v0.35, FASTQC v0.11.4, metaSPAdes (SPAdes 3.7.0), Prodigal v2.60, MAFFT v7.471, trimAl v1.4.rev15, IQTREE v1.6.9, v16.12, v2.0, KALLISTO v0.42.5, CONCOCT v0.4.0, MMGENOME (as on GitHub june 2016), Bowtie2 v2.3, CLARK-S v1.2.3, miComplete v.1.1.1, prokka v1.12,<br>eggNOG-mapper v1.0.3, GhostKOALA (KEGG tools) v2.2, HMMER v3.3, InterProScan v5.42-78.0, MacSyFinder v2.0rc1, DIAMOND v2.0.6.144, PSI-BLAST<br>v2.8.1+, BMGE v1.12, PREQUAL v1.01, Divvier v1.01, PhyloBayes MPI v1.8, ALE v0.4, BLASTN v2.8.1+, BLASTP v2.8.1+<br><br>Custom code:<br>https://github.com/maxemil/rickettsiales-evolution,<br>https://github.com/maxemil/ALE-pipeline,<br>https://github.com/novigit/broCode

For manuscripts utilizing custom algorithms or software that are central to the research but not yet described in published literature, software must be made available to editors and reviewers. We strongly encourage code deposition in a community repository (e.g. GitHub). See the Nature Portfolio guidelines for submitting code & software for further information.

Data

Policy information about availability of data

All manuscripts must include a data availability statement. This statement should provide the following information, where applicable:

- Accession codes, unique identifiers, or web links for publicly available datasets
- A description of any restrictions on data availability
- For clinical datasets or third party data, please ensure that the statement adheres to our policy

In addition to data available in the supplementary materials, files containing sequence datasets, alignments, and phylogenetic trees in Newick format are archived at the digital repository Figshare: 10.6084/m9.figshare.c.5494977. MAGs generated in this study are linked to BioProject PRJNA746308. Accessions for genomes analyzed in this study can be found in Supplementary Data 3. Publicly available datasets include eggNOG v4.5.1 (eggnog45.embl.de/), KEGG (kegg.jp), CAZY (cazy.org), TCDB (tcdb.org), PFAM (pfam.xfam.org), TIGRFAM and InterPro (ebi.ac.uk/interpro/), NCBI nucleotide (ncbi.nlm.nih.gov/nucleotide/), MetaCyc v.26.0 (biocyc.org/META/)

Field-specific reporting

Please select the one below that is the best fit for your research. If you are not sure, read the appropriate sections before making your selection.

☐ Life sciences ☐ Behavioural & social sciences ☒ Ecological, evolutionary & environmental sciences

For a reference copy of the document with all sections, see nature.com/documents/nr-reporting-summary-flat.pdf

Ecological, evolutionary & environmental sciences study design

All studies must disclose on these points even when the disclosure is negative.

Study description	Reconstruction of novel alphaproteobacterial genomes (MAGs). Phylogenomic analyses of Rickettsiales and related MAGs. Gene-tree species tree reconciliation and ancestral reconstruction of the last common ancestor of Rickettsiales.
Research sample	Available metagenomic datasets from the Tara Oceans consortium, published MAGs and reference genomes of Rickettsiales and alphaproteobacteria
Sampling strategy	We selected particular metagenomic datasets of the Tara Oceans consortium and the other MAGs based on a phylogenetic screen of contigs containing ribosomal protein genes. Those datasets and MAGs that contained contigs related to Rickettsiales were selected
Data collection	N/A because the primary data collection was done by other parties (i.e. the Tara Oceans expedition and other research groups that collected the raw sequence data underlying the MAGs we selected)
Timing and spatial scale	N/A because we did not do the primary data collection
Data exclusions	Certain Rickettsiales taxa were excluded from phylogenetic analyses as they were found to have extremely long branches and their inclusion would lead to untrustworthy results due to long branch attraction artefacts. Criteria for excluding were not pre-established
Reproducibility	All results of this study can be reproduced given the same original source data and the methods provided in this manuscript
Randomization	N/A because randomization was not required for the purposes of this study
Blinding	N/A because blinding was not required for the purposes of this study

Did the study involve field work? ☐ Yes ☒ No

Reporting for specific materials, systems and methods

We require information from authors about some types of materials, experimental systems and methods used in many studies. Here, indicate whether each material, system or method listed is relevant to your study. If you are not sure if a list item applies to your research, read the appropriate section before selecting a response.

Materials & experimental systems

n/a	Involved in the study
☒	☐ Antibodies
☒	☐ Eukaryotic cell lines
☒	☐ Palaeontology and archaeology
☒	☐ Animals and other organisms
☒	☐ Human research participants
☒	☐ Clinical data
☒	☐ Dual use research of concern

Methods

n/a	Involved in the study
☒	☐ ChIP-seq
☒	☐ Flow cytometry
☒	☐ MRI-based neuroimaging
